# The effect of litter hierarchy and teat allocation on suckling piglets' growth

**DOI:** 10.5194/aab-68-263-2025

**Published:** 2025-04-09

**Authors:** Fernando Mata, José Araújo, Alicja Kowalczky, Joaquim Cerqueira

**Affiliations:** 1 Center for Research and Development in Agrifood Systems and Sustainability, Instituto Politécnico de Viana do Castelo, Rua Escola Industrial e Comercial Nun'Álvares, 34, 4900-347 Viana do Castelo, Portugal; 2 Escola Superior Agrária, Instituto Politécnico de Viana do Castelo, Refóios do Lima, 4990-706 Ponte de Lima, Portugal; 3 Mountain Research Centre, Instituto Politécnico de Viana do Castelo, Rua da Escola Industrial e Comercial Nun'Alvares 34, 4900-347 Viana do Castelo, Portugal; 4 Department of Environmental Hygiene and Animal Welfare, Wrocław University of Environmental and Life Sciences, Chełmońskiego 38c, 51-630 Wrocław, Poland; 5 Veterinary and Animal Research Centre (CECAV), University of Trás-os-Montes and Alto Douro, Quinta de Prados, Apartado 1013, 5001-801 Vila Real, Portugal

## Abstract

The objective of the current study was to adjust a growing curve to suckling piglets as a function of the hierarchical position achieved and of mammary gland allocation (anterior, medium, or posterior) for the investigation of, eventually, different growing patterns. For this purpose, 280 piglets from 20 sows (14 per sow) were weighed and observed from birth to weaning. The birth weight of piglets in the three groups was found to be significantly different ( P<0.05 ), with heavier piglets gaining access to the more productive anterior teats. The quadratic curve was found to be the best fit to model piglets' growth up to weaning. Higher hierarchical positions chose the most productive mammary glands in decreasing order: the anterior, medium, and posterior areas of the venter of the sow. Piglets in the different teat-suckling groups (anterior, medium, and posterior) studied have significantly different growing patterns. The anterior-teat-suckling piglets' growth curve can be differentiated almost immediately from the beginning and up to weaning from the medium- and the posterior-teat-suckling groups. These last two teat-suckling groups (medium and posterior) can be differentiated from day 20 and up to weaning. At weaning (28 d), the three distinct groups have significantly different weights ( P<0.001 ). Piglets' birth weights correlate positively with a higher hierarchy. A higher hierarchy results in heavier weaning once the piglets gain access to the more productive teats. The number of functional teats in relation to larger litter sizes needs attention from pig breeders to mitigate welfare issues while maintaining reproductive performance.

## 1 Introduction

Domestic pigs occupy a large part of the animal production systems around the world. Therefore, genetic improvement programmes have been directed toward increased profitability through prolificacy (Chen et al., 2001; Rohrer and Nonneman, 2017). The total number of born piglets per sow in Denmark increased from 11.6 in 2000 to 15.7 in 2021 (Knap et al., 2023). Larger litter sizes of 18 piglets were common in Denmark and (despite a change in the selection policy in 2004) were implemented to decrease early mortality and to redirect selection toward survivability at day 5; however, the mortality rates observed at farrowing and lactation were still 9.4 % and 13.5 %, respectively (Christensen et al., 2018).

Larger litter sizes result in increased fighting between siblings to access the more productive teats of the sow. The more productive teats are positioned (in decreasing order) in the anterior (pectoral or thoracic), medium (abdominal), and posterior (inguinal) ventral areas of the sow (Farmer et al., 2017; Skok et al., 2007). There are several key pieces of research on teat hierarchy (e.g. De Passillé and Rushen, 1989a; Rosillon-Warnier and Paquay, 1984). These authors determined that there was a progressive trend in teat order development, with piglets initially directing themselves toward teats near the angles formed by the body and legs of the sow and with piglets also showing a high tendency to move toward the front. The authors concluded that piglets suckling from anterior teats gain weight faster than the others. De Passillé and Rushen (1989b) determined the cyclic suckling behaviour of piglets and found that the anterior teats were preferable, which was indicated by additional fighting and suckling. Teat hierarchy is affected by litter size, and piglets who win disputes suckle with increased frequency, developing fidelity to a specific teat (Chou et al., 2022).

The characteristics of the litter have an influence on the sow's milk production, namely the nursing frequency, the litter size, and the pig size. Nursing intervals, which typically range from 30 to 70 min, influence milk yield. Frequent and complete gland emptying helps regulate milk production by preventing inhibitory feedback caused by milk accumulation. Conditioning sows to nurse in response to cues or auditory stimuli can optimize milk output (Auldist et al., 2000). Strategies that encourage frequent nursing and complete milk extraction are essential for maximizing milk yield. Auldist et al. (1998) showed a positive correlation between milk yield and litter size, with milk yields increasing as litter sizes ranged from 4 to 14 piglets. While early lactation showed no clear plateau in milk yield, late lactation tended to reach a maximum at larger litter sizes. Individual piglet milk intake decreases with increasing litter size due to greater competition, but overall milk production per sow rises as the number of functional glands increases. King et al. (1997) showed that heavier piglets stimulate greater milk production as they can extract more milk from the sow. Boyce et al. (1997) found that litters containing piglets 0.4 kg heavier grew more quickly before weaning. Fostering newborn piglets onto sows already in lactation can reduce milk yield by 22 %, whereas fostering older piglets onto newly farrowed sows can increase yield by 20 %. Heavier piglets stimulate better blood flow and hormone release (e.g. oxytocin and prolactin) during nursing. The prediction of pigs' weights throughout their growth can be achieved with mathematical models. These models provide a tool for management decisions and are, therefore, advantageous to producers (Schinckel and De Lange, 1996) (e.g. in the estimation of daily protein accretion rates for phase, split-sex, and split-breed feeding systems). As a result, several growth equations have been used to model pigs' growth and to relate weight with age. Several empirical growth equations have been used to model the sigmoidal shape of pigs' growth, such as the Gompertz, the logistic, the Richards, and the von Bertalanffy equations (Wellock et al., 2004).

The most common parameterization of these growth curves can be seen in Table 1. These curves can be obtained through the change of the parameter d in the Richards equation (Tjørve and Tjørve, 2010), expressed by the equation

1 $$W\left(t\right)=\left(a^{1-m}-b\cdot \mathrm{exp}{\left(-ct\right)}^{1-1-m}\right),$$

where the parameters have the following biological interpretation: a is mature body weight, b represents the constant of integration (no biological interpretation), c is the maturity rate, and m is the inflection parameter (Beltran et al., 1992). The maturity rate represents the rate at which the animal approaches its mature weight (Crispim et al., 2015).

These curves express weight gain as a function of time and differ in terms of the parameters of their equations. However, in the current study, the growing period was considered up to the weaning age only. During this stage, piglets grow with daily positive increments, and, therefore, the sigmoidal shape does not apply, and another type of curve can fit the data better. Renner-Martin et al. (2016) have compared different curves to model weight gain in piglets and found that, before weaning, the best fits are obtained with the von Bertalanffy, quadratic, and cubic curves.

**Table 1:** Parameterizations of common equations used to model pigs' growth.

Model	Equation
Gompertz	$W\left(t\right)=a(\mathrm{exp}(-b\mathrm{exp}\left(-ct\right))$
Logistic	$W\left(t\right)=a(1+b\mathrm{exp}(-ct){)}^{-1}$
von Bertalanffy	$W\left(t\right)=a(1-b\mathrm{exp}(-ct){)}^{3}$
Richards	$W\left(t\right)=a(1-b\mathrm{exp}(-ct){)}^{d}$

Note that W(t) refers to weight at time t , and a , b , and d are parameters of the equations.

Previous studies have been more concentrated on the development of teat order alone, with only a slight relation to its effect on piglet growth. Placing detailed information into a growth curve and correlating both teat order and weight gain would aid in the understanding of the extent of the impact of teat hierarchy, thus allowing appropriate measures to be predicted and taken for productive success. The first objective of the present study was to find the best model to adjust to piglet growth up to weaning. The second objective was to adjust the model to the growth period of piglets allocated to different suckling teats (anterior, medium, and posterior). The final aim of this study was to evaluate the impact of teat hierarchy on the weaning weight of the piglets. As birth weight may directly influence the hierarchical allocation of teats to the piglets and may also be directly correlated with the weight at weaning, we have also tested these hypotheses.

## 2 Material and methods

### 2.1 Sampling

The study was conducted at the farm Quinta da Beira, Castelo Branco, Portugal, from 2 May to 29 June 2021. Quinta da Beira is a pig farm with 240 breeding sows, which are kept in a group housing system with straw bedding. Sows enter a farrowing crate only during lactation and up to piglets' weaning. The weaned piglets are grown and fattened in group housing with straw bedding in split-phase and split-sex feeding. Pigs are slaughtered at 6 months of age, with approximately 110–120 kg of live weight.

To achieve the study aim, weight measurements were recorded for 20 piglets born from Large White × Landrace sows, sired with Duroc boars. All records were taken at the same farm, in the same farrowing room, and in the same type of farrowing crate. The room temperature was set to 22 °C, and the litter mat temperature was set to 32 °C. Sows were in parity 4 to 6 and were left with 14 piglets each after farrowing. Several sows farrowed more than 14 piglets, and, in this case, the extra piglets were cross-fostered to other sows not in the study groups. Sows farrowing less than 14 piglets were not considered in the study. All the sows in the study had at least 14 functional mammary glands. It was left to the discretion of the farmer to choose the piglets to be removed for cross-fostering and/or hand-rearing.

A total of N=280 piglets were weighted and observed for teat hierarchy from their birthday (day 1) to weaning (day 28). Weights were recorded every day in the first 7 d and then every 5 d until weaning (days 13, 18, 23, and 28). Teat order recordings were also taken during the first 7 d while the hierarchy was being established. The piglets were marked with colour crayons for recognition and were observed directly in the morning for approximately 15 min per sow during suckling time. Teat sampling was more evident on the first, second, and third day after farrowing. After establishing the teat hierarchy, the piglets were allocated to one of the groups being studied (anterior- or pectoral-, medium- or abdominal-, or posterior- or inguinal-teat sucklers) for growth comparison between suckling groups. The sex of the piglets was not considered in this study as it does not affect either birth weight or weaning weight (Škorjanc et al., 2007). The models that were previously identified as the best fit for this period of growth were the von Bertalanffy, quadratic, and cubic curves (Renner-Martin et al., 2016). We have, therefore, used these curves to find the best-fit model in our study.

### 2.2 Statistical analysis

The statistical analysis and the curve fitting were performed using the freeware R CRAN for Windows^®^ version 4.0.4 platform x86_64-w64-mingw32/x64 (64 bit) (Comprehensive R Archive Network, http://cran.r-project.org/, last access: 1 September 2024). The graph was produced with Microsoft Excel^®^ for Windows^®^.

For the test of the eventual significant differences in the birth and weaning weights of the three groups of piglets, we used a fixed-effect analysis of variance (ANOVA), with the least significant difference (LSD) test used post hoc. The residuals were tested for normality with the Kolmogorov–Smirnov test. We set P<0.05 as the significance level in all the tests.

Mean weights and standard deviations (SDs) were calculated on day 1; every day up to day 7; and then on days 13, 18, 23, and 28 for each of the suckling groups (anterior-, medium-, or posterior-teat sucklers). The SDs were used to calculate 95 % confidence intervals (CIs). The series of mean weights for given days, together with the upper and lower CIs, were then used to fit quadratic, cubic, and von Bertalanffy curves. It was found that the best fit, with the higher coefficient of determination ( $R^{2}=0.99$ ), was obtained by both the cubic and the quadratic curves. Therefore, the quadratic curve was chosen to model the data as it is the most parsimonious model. The equations of the models fitted to both the means and the CIs were then used to produce the growth curves of the three groups of piglets, together with 95 % confidence envelopes (CEs) connecting all the upper and lower confidence intervals for a given time. The intersection of the 95 % CE was then analysed to evaluate significantly different growth rates between the suckling groups from day 1 (birth) up to day 28 (weaning).

## 3 Results and discussion

The ANOVA test for the different suckling groups shows a significantly different ( P=0.028 ) birth weight of the piglets (Table 1). The ANOVA test of weaner piglets' weights of the three suckling groups also shows significant differences ( P<0.001 ) (Table 2). The residuals for both ANOVAs were found to be normally distributed ( P>0.05 ).

**Table 2:** Significant differences ( P<0.001 ) in birth and weaning weights within the groups of suckling piglets being studied. One-way ANOVA employed, with the test for least significant differences used post hoc.

	Suckling group		
Weight (kg)	Anterior	Medium	Posterior
Birth	1.67^a^	1.55^b^	1.42^c^
Weaning	7.83^a^	6.93^b^	5.99^c^

Note that different superscripted letters are indicative of significant differences after the LSD test ( P<0.05 ).

Teat sampling, or the action taken by the piglets to try different teats, began immediately after birth, and hierarchies began to establish as a function of the higher or lower production and release of milk in the different teats. The teat order is established when the piglets are allocated to one teat and do not move to different teats. In this study, the teat order was fully established by the end of 7 d, and the piglets were then recorded as suckling the anterior (two pairs of pectoral teats), medium (three or four pairs of abdominal teats), or posterior (two pairs of inguinal teats) teats. Some sows have seven pairs of functional teats, while others have eight pairs. In either case, the last two functional pairs (inguinal) are classified as the posterior. A total of n=80 , n=137 , and n=63 piglets were recorded in each group of piglets suckling the anterior, the medium, and the posterior teats, respectively.

**Figure 1:**
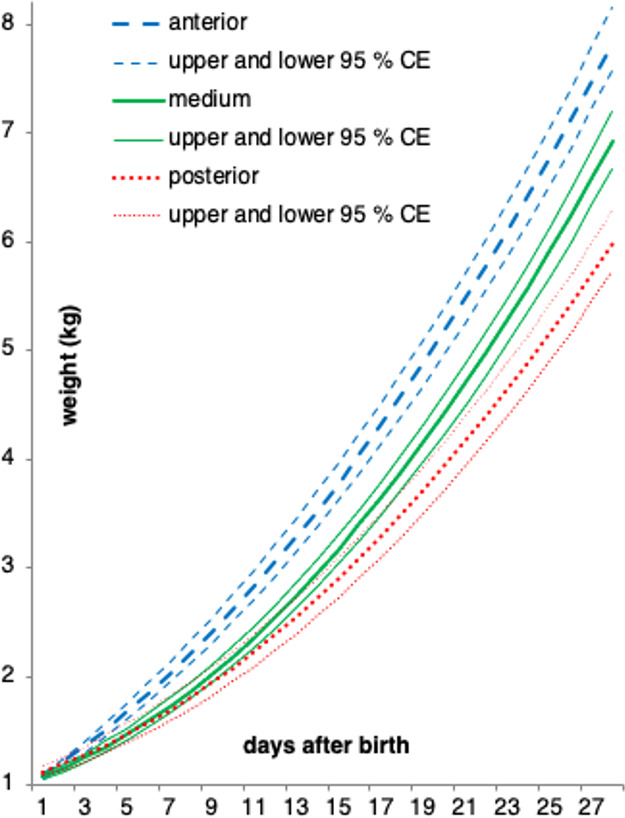
Quadratic curves and respective 95 % confidence envelopes (CEs) used to model the three groups of teat-suckling piglets being studied (anterior, medium, posterior).

The equation parameters of the curves fitted to the three suckling groups of piglets, together with the respective 95 % CEs, are presented in Table 3. In Fig. 1, we can see the graphical representation of these curves and the respective 95 % CEs.

The group suckling the anterior teats differs from the others almost immediately from the beginning (CEs fail to intersect from approximately day 4). The curves representing the lower and upper 95 % CEs of the medium- and the posterior-suckling groups, respectively, fail to intersect from day 18. Therefore, from day 18 onward, the weight between these two groups differs as well.

Within the first 2–6 h after farrowing, newborn piglets undergo teat sampling, where they will sample each teat to determine which they prefer (Algers, 1993; Vitali et al., 2020). Once sampling is complete, a teat order begins to develop amongst the sibling piglets in the litter and will be completely established within 7 d, remaining throughout the suckling period (Skok et al., 2007). More dominant piglets suckle from the anterior teats as these have the highest milk production, whereas the others are likely to occupy the middle or posterior teats, which have lower milk production levels (Vitali et al., 2020). Note that the higher levels of production in anterior teats are independent of an eventual higher degree of stimulation provided by piglets with higher weights (Skok et al., 2007). Piglets which have established fidelity to a particular teat are more likely to survive (De Passillé et al., 1988), whereas the others may die or only survive by opportunistically suckling (Milligan et al., 2002). Newborn piglets possess erupted canines that are slightly sideward orientated, providing them with the capacity for side biting when in competition with litter siblings, and most of the observable lesions in piglets' faces are caused by this fierce competition (Vitali et al., 2020).

**Table 3:** Parameters of the quadratic curves and respective 95 % confidence envelopes (CEs) used to model the three groups of suckling piglets being studied.

Group	Parameter curve	a	b	c
Anterior	Upper 95 % CE	0.0044	0.1422	1.1095
	Mean	0.0044	0.1312	1.0802
	Lower 95 % CE	0.0045	0.1202	1.0509
Medium	Upper 95 % CE	0.0053	0.0825	1.1050
	Mean	0.0052	0.0764	1.0774
	Lower 95 % CE	0.0051	0.0703	1.0498
Posterior	Upper 95 % CE	0.0040	0.0807	1.1784
	Mean	0.0041	0.0695	1.1221
	Lower 95 % CE	0.0042	0.0584	1.0659

Note that, for the equations, weight (kg) = $a^{2}x+bx+c$ , where x is the day. For all the curves, the coefficient of determination $R^{2}=0.99$ .

In agreement with the results of the current study, other authors such as Beaulieu et al. (2010) and Jankowiak et al. (2020) have also reported that piglets with higher birth weights are heavier at weaning. The reduction in birth weight is correlated with factors such as litter size and sow nutrition (Moreira et al., 2020). Intrauterine crowding caused by large litter sizes causes an insufficient supply of both nutrients and oxygen to the embryos in development due to placenta size limitations (Foxcroft et al., 2009). The reduction in growth rate can be explained by a reduction in the number of muscle fibres at birth (Bérard et al., 2011). As muscle growth is achieved by hypertrophic enlargement of the existing muscle fibres, the growth rate after birth is determined by the birth weight (Stange et al., 2020).

The location and number of mammary glands in mammals vary, showing, normally, bilateral symmetry and low variation in the number of functional teats (Propper et al., 2013). Pigs, however, are an exception to this generic rule, which also applies to the location across the complete ventral area (anterior or pectoral, medium or abdominal, and posterior or inguinal) (Rohrer and Nonneman, 2017). The breeding programmes of pigs have concentrated attention on increasing litter size and the number of functional mammary glands (Andersen et al., 2011); however, the litter size has increased faster than the number of functional mammary glands (Earnhardt and Knauer, 2019; Li et al., 2021; Speckman et al., 2021). The number of functional mammary glands averages below 15 (Dall'Olio et al., 2018), while, in the last 20 years, the litter size has increased (with Danish sows) from 11.7 live piglets (in the year 2000) to 17.5 live piglets (in the year 2019) (Sell-Kubiak, 2021). The result, therefore, is increased intrauterine crowding, low birth weights, and higher pre-weaning mortality by starvation (Andersen et al., 2011).

The weaning weight of pigs has been related to faster post-weaning growth (Faccin et al., 2020), life performance (Collins et al., 2017), and the production of better carcasses at slaughter (Wolter and Ellis, 2001). Therefore, aiming for homogeneity between birth weights and functional mammary glands that are capable of providing for the litter homogeneously would be beneficial; otherwise, the welfare of the piglets and sows is compromised (Oliviero et al., 2019; Peltoniemi et al., 2020). The development of competitive behaviours between piglets can also eventually impact their future behaviour in the growing and finishing phases, with suggestions having been made that increased competition during the suckling period may contribute to increased tail-biting behaviour in later stages (Vitali et al., 2020).

The advances in genetics with regard to litter size and functional mammary glands may raise welfare concerns. Several studies (e.g. Earnhardt and Knauer, 2019; Obermier et al., 2023; Speckman et al., 2021; Wiegert and Knauer, 2018) have demonstrated a negative correlation between the number of functional mammary glands and the mortality rates in pre-weaning in piglets. Efforts should be made to reduce mortality, which may be achieved by means of genetic selection for homogeneous birth weights between litter mates and for litter sizes that are concordant with the number of homogeneously productive mammary glands. The number of functional mammary glands is a heritable trait, and the selection advancement of these traits can be tracked through time with genetic markers (Rohrer and Nonneman, 2017). Identifying genes responsible for controlling the number of functional mammary glands deserves the attention of researchers (Li et al., 2021). The number of functional teats in sows has the potential to be increased based on Chinese breeds' mutation genes, with sows carrying up to 20 functional mammary glands (Yang et al., 2016).

To achieve good productive and welfare standards, cross-fostering and hand-rearing remain as alternatives, while tooth clipping, despite being legal, raises welfare questions. Nevertheless, the limitations imposed by the interaction between litter size and the number of functional mammary glands are a major concern in pig welfare (Knap et al., 2023; Vitali et al., 2020).

This study did not include factors such as breed, farming system, environmental conditions, and specialist nutrition programmes; therefore, these limitations provide avenues for future research.

In conclusion, the quadratic curve fits very well with the suckling growth phase of piglets. Teat order is established depending on birth weight and influences weight gain throughout the pre-weaning period. Birth weight influences access to more productive teats, and, therefore, piglets with larger birth weights take hold of and gain fidelity to the more productive teats in the anterior area of the sow venter and end up being weaned more heavily. The number of functional teats in relation to the litter size remains a welfare issue deserving attention.

## Data Availability

The datasets generated are available from the corresponding author on request.

## References

[bib1.bib1] Algers B (1993). Nursing in pigs: communicating needs and distributing resources. J Anim Sci.

[bib1.bib2] Andersen IL, Nævdal E, Bøe KE (2011). Maternal investment, sibling competition, and offspring survival with increasing litter size and parity in pigs (Sus scrofa). Behav Ecol Sociobiol.

[bib1.bib3] Auldist DE, Morrish L, Eason P, King RH (1998). The influence of litter size on milk production of sows. Anim Sci.

[bib1.bib4] Auldist DE, Carlson D, Morrish L, Wakeford CM, King RH (2000). The influence of suckling interval on milk production of sows. J Anim Sci.

[bib1.bib5] Beaulieu AD, Aalhus JL, Williams NH, Patience JF (2010). Impact of piglet birth weight, birth order, and litter size on subsequent growth performance, carcass quality, muscle composition, and eating quality of pork. J Anim Sci.

[bib1.bib6] Beltran JJ, Butts Jr. WT, Olson TA, Koger M (1992). Growth patterns of two lines of Angus cattle selected using predicted growth parameters. J Anim Sci.

[bib1.bib7] Bérard J, Kalbe C, Lösel D, Tuchscherer A, Rehfeldt C (2011). Potential sources of early-postnatal increase in myofibre number in pig skeletal muscle. Histochem Cell Biol.

[bib1.bib8] Boyce JM, Smits RJ, Campbell RG, King RH (1997). The interrelationship between parity and litter weight on the milk production of sows. In Proc VI Conf Aust Pig Sci Assoc.

[bib1.bib9] Chen P, Baas TJ, Dekkers JCM, Christian LL (2001). Selection for lean growth rate and correlated responses in litter traits in a synthetic line of Yorkshire-Meishan pigs. Can J Anim Sci.

[bib1.bib10] Chou JY, Marchant JN, Nalon E, Huynh TTT, Van de Weerd HA, Boyle LA, Ison SH (2022). Investigating risk factors behind piglet facial and sow teat lesions through a literature review and a survey on teeth reduction. Front Vet Sci.

[bib1.bib11] Christensen OF, Kongsted H, Lund MS (2018). Evaluering af avl for LG5, Aarhus, DCA – Nationalt Center for Fødevarer og Jordbrug. https://pure.au.dk/ws/portalfiles/portal/128087355/LG5_evaluering010618.pdf.

[bib1.bib12] Collins CL, Pluske JR, Morrison RS, McDonald TN, Smits RJ, Henman DJ, Stensland I, Dunshea FR (2017). Post-weaning and whole-of-life performance of pigs is determined by live weight at weaning and the complexity of the diet fed after weaning. Anim Nutr.

[bib1.bib13] Crispim AC, Kelly MJ, Guimarães SEF, e Silva FF, Fortes MRS, Wenceslau RR, Moore S (2015). Multi-trait GWAS and new candidate genes annotation for growth curve parameters in Brahman cattle. PLoS One.

[bib1.bib14] Dall'Olio S, Ribani A, Moscatelli G, Zambonelli P, Gallo M, Costa LN, Fontanesi L (2018). Teat number parameters in Italian Large White pigs: Phenotypic analysis and association with vertnin (VRTN) gene allele variants. Livest Sci.

[bib1.bib15] De Passillé AMB, Rushen J (1989). Suckling and teat disputes by neonatal piglets. Appl Anim Behav Sci.

[bib1.bib16] De Passillé AMB, Rushen J (1989). Using early suckling behavior and weight gain to identify piglets at risk. Can J Anim Sci.

[bib1.bib17] De Passillé AMB, Rushen J, Hartsock TG (1988). Ontogeny of teat fidelity in pigs and its relation to competition at suckling. Can J Anim Sci.

[bib1.bib18] Earnhardt AL, Knauer M (2019). PSII-30 The genetics of functional teats in swine. J Anim Sci.

[bib1.bib19] Faccin JEG, Laskoski F, Cemin HS, Mellagi APG, Bernardi ML, Ulguim RR, Bortolozzo FP, Tokach MD (2020). Evaluating the impact of weaning weight and growth rate during the first week post weaning on overall nursery performance. J Swine Health Prod.

[bib1.bib20] Farmer C, Fortin E, Méthot S (2017). In vivo measures of mammary development in gestating gilts. J Anim Sci.

[bib1.bib21] Foxcroft GR, Dixon WT, Dyck MK, Novak S, Harding JCS, Almeida FCRL (2020). Prenatal programming of postnatal development in the pig. CPRCPR28.

[bib1.bib22] Jankowiak H, Balogh P, Cebulska A, Vaclavkova E, Bocian M, Reszka P (2020). Impact of piglet birth weight on later rearing performance. Vet Med Czech.

[bib1.bib23] King RH, Mullan BP, Dunshea FR, Dove H (1997). The influence of piglet body weight on milk production of sows. Livest Prod Sci.

[bib1.bib24] Knap PW, Knol EF, Sørensen AC, Huisman AE, van der Spek D, Zak LJ, Granados Chapatte A, Lewis CRG (2023). Genetic and phenotypic time trends of litter size, piglet mortality, and birth weight in pigs. Front Anim Sci.

[bib1.bib25] Li Y, Pu L, Shi L, Gao H, Zhang P, Wang L, Zhao F (2021). Revealing New Candidate Genes for Teat Number Relevant Traits in Duroc Pigs Using Genome-Wide Association Studies. Animals.

[bib1.bib26] Milligan BN, Fraser D, Kramer DL (2002). Within-litter birth weight variation in the domestic pig and its relation to pre- weaning survival, weight gain, and variation in weaning weights. Livest Prod Sci.

[bib1.bib27] Moreira RHR, Perez Palencia JY, Moita VHC, Caputo LSS, Saraiva A, Andretta I, Ferreira RA, de Abreu MLT (2020). Variability of piglet birth weights: A systematic review and meta‐analysis. J Anim Physiol Anim Nutr.

[bib1.bib28] Obermier DR, Howard JT, Gray KA, Knauer MT (2023). The impact of functional teat number on reproductive throughput in swine. Transl Anim Sci.

[bib1.bib29] Oliviero C, Junnikkala S, Peltoniemi O (2019). The challenge of large litters on the immune system of the sow and the piglets. Reproduction in Domestic Animals.

[bib1.bib30] Peltoniemi O, Oliviero C, Yun J, Grahofer A, Björkman S (2020). Management practices to optimize the parturition process in the hyperprolific sow. J Anim Sci.

[bib1.bib31] Propper AY, Howard BA, Veltmaat JM (2013). Prenatal morphogenesis of mammary glands in mouse and rabbit. J Mammary Gland Biol.

[bib1.bib32] Renner-Martin K, Kühleitner M, Brunner N, Hagmüller W (2016). AIC-Based Selection of Growth Models: The Case of Piglets from Organic Farming. Open Journal of Modelling and Simulation.

[bib1.bib33] Rohrer GA, Nonneman DJ (2017). Genetic analysis of teat number in pigs reveals some developmental pathways independent of vertebra number and several loci which only affect a specific side. Genet Sel Evol.

[bib1.bib34] Rosillon-Warnier A, Paquay R (1984). Development and consequences of teat-order in piglets. Appl Anim Behav Sci.

[bib1.bib35] Schinckel AP, De Lange CFM (1996). Characterization of growth parameters needed as inputs for pig growth models. J Anim Sci.

[bib1.bib36] Sell-Kubiak E (2021). Selection for litter size and litter birthweight in Large White pigs: Maximum, mean and variability of reproduction traits. Animal.

[bib1.bib37] Skok J, Brus M, Škorjanc D (2007). Growth of piglets in relation to milk intake and anatomical location of mammary glands. Acta Agr Scand Sect A.

[bib1.bib38] Škorjanc D, Brus M, Čandek Potokar M (2007). Effect of Birth Weight and Sex on Pre-Weaning Growth Rate of Piglets. Arch Anim Breed.

[bib1.bib39] Speckman EC, Howard JT, Wiegert JG (2021). The Relationship Between Litter Size and Functional Teat Number at Farrowing on Litter Size at Weaning. J Anim Sci.

[bib1.bib40] Stange K, Miersch C, Sponder G, Röntgen M (2020). Low birth weight influences the postnatal abundance and characteristics of satellite cell subpopulations in pigs. Sci Rep.

[bib1.bib41] Tjørve E, Tjørve KM (2010). A unified approach to the Richards-model family for use in growth analyses: why we need only two model forms. J Theor Biol.

[bib1.bib42] Vitali M, Santacroce E, Correa F, Salvarani C, Maramotti FP, Padalino B, Trevisi P (2020). On-farm welfare assessment protocol for suckling piglets: a pilot study. Animals.

[bib1.bib43] Wellock IJ, Emmans GC, Kyriazakis I (2004). Describing and predicting potential growth in the pig. Anim Sci.

[bib1.bib44] Wiegert JG, Knauer MT (2018). 98 Sow Functional Teat Number Impacts Colostrum Intake and Piglet Throughput. J Anim Sci.

[bib1.bib45] Wolter BF, Ellis M (2001). The effects of weaning weight and rate of growth immediately after weaning on subsequent pig growth performance and carcass characteristics. Can J Anim Sci.

[bib1.bib46] Yang J, Huang L, Yang M, Fan Y, Li L, Fang S, Deng W, Cui L, Zhang Z, Ai H (2016). Possible introgression of the VRTN mutation increasing vertebral number, carcass length and teat number from Chinese pigs into European pigs. Sci Rep.

